# Collagen changes in rabbit conjunctiva after conjunctival crosslinking

**DOI:** 10.1515/biol-2022-0604

**Published:** 2023-05-23

**Authors:** Li-Juan Mo, Han-Min Wang, Huan-Ming Zhou, Li Huang, Yan-Xiang Gui, Qing-Song Li

**Affiliations:** Department of Ophthalmology, Putuo Hospital, Shanghai University of Traditional Chinese Medicine, No. 164 Lanxi Road, Putuo District, Shanghai 200062, China; Department of Gerontology, Putuo Hospital, Shanghai University of Traditional Chinese Medicine, Shanghai, China

**Keywords:** conjunctiva, conjunctival crosslinking, collagen, rabbit

## Abstract

This study aims to determine the ultrastructural changes in collagen fibrils in rabbit conjunctiva after conjunctival crosslinking using riboflavin and ultraviolet A (UVA) light at an irradiation intensity of 45 mW/cm^2^. Conjunctival crosslinking may increase conjunctival stiffness. The supertemporal quadrants of the right eyes of 24 adult rabbits were treated with a topical riboflavin solution (0.25%) before irradiation with UVA light at 45 mW/cm^2^ for 4 min. After 3 weeks, the collagen fibrils in fibril bundles were examined by electron microscopy. Immunohistochemical staining was used to detect the expression levels of collagen I and collagen III in the rabbits’ conjunctiva. The diameter of the collagen fibrils in the fibril bundles varied slightly, ranging from 30 to 60 nm in the conjunctival stroma of the control group. In the treatment group, the diameter of collagen fibrils ranged from 60 to 90 nm. The thickest collagen fibrils were observed in the treatment group (up to 90 nm in diameter). In contrast, those in the conjunctival stroma of the control group were considerably smaller (up to 60 nm in diameter). However, thicknesses of collagen fibrils displayed a unimodal distribution. Both collagen I and collagen III increased after treatment with riboflavin and UVA light irradiation at 45 mW/cm^2^. The data indicate that in rabbits, conjunctival crosslinking with riboflavin and UVA light at 45 mW/cm^2^ for 4 min is safe and does not induce ultrastructural alterations of the conjunctival cells. The conjunctival crosslinking with riboflavin and UVA light at 45 mW/cm^2^ can increase the diameter of collagen fibrils, but the average densities of collagen I and collagen III have no statistical significance.

## 1 Introduction

Conjunctivochalasis (CCh) is a common eye disease related to age characterized by loosely redundant conjunctiva folds between the eyeball and the lower eyelid, causing a foreign body sensation, tears, and other symptoms [1]. The condition is associated with the biomechanical properties of the conjunctiva [1,2]. During CCh development, the conjunctiva undergoes an active remodeling process, which causes a progressive thinning and weakening of the tissue. A reduction in the size and number of the collagen fibers of the conjunctiva is known to be the main pathogenic factor in progressive CCh, during which the conjunctiva becomes weaker and more extensible [2].

Despite extensive research, there is still no effective method to prevent the progression of CCh. Recently, riboflavin and ultraviolet A (UVA)-induced collagen crosslinking (CXL) was successfully used to prevent the progression of keratoconus and keratectasia after photorefractive keratectomy and laser *in situ* keratomileusis (LASIK) [3,4]. In 2016, Choy et al. [5] conducted a prospective study on five patients and concluded that a single session of crosslinking was effective in limiting leakage from the filtering bleb. In recent years, more scholars have also confirmed that CXL could strengthen collagen fibers of the conjunctiva that form the outer lining of the cystic bleb and thus would be effective in treating bleb leak [6,7]. Therefore, for typically collagenous tissues like the cornea, it can be logically hypothesized that conjunctival CXL may strengthen the conjunctiva to prevent CCh progression.

Based on this hypothesis, we conducted an electron microscopy and immunohistochemical (IHC) staining investigation aiming to provide a comprehensive ultrastructural description and a morphometric analysis of the conjunctival structure to explore the CXL technology used in CCh.

## 2 Materials and methods

All methods are reported in accordance with ARRIVE guidelines.

### 2.1 Animals and anesthesia

Twenty-four adult New Zealand white rabbits (aged 3–4 months) were used in this experiment. The animals were maintained with free access to water and food in an air-conditioned room on a 12-h light–dark cycle. The animals were anesthetized with intramuscular ketamine (50 mg/kg) plus xylazine (10 mg/kg). To prevent a decrease in body temperature, the animals were positioned on a heated workstation. Conjunctival crosslinking was performed in the right eyes of the animals; the left eyes remained untreated and served as the control group.


**Ethical approval:** The research related to animal use has been complied with all the relevant national regulations and institutional policies for the care and use of animals and has been approved by the Ethics Committee of Putuo District Center Hospital, Shanghai (Putuo Hospital, Shanghai University of Traditional Chinese Medicine) (No: PTEC-R-2020-23(Y)-1).

### 2.2 Conjunctival crosslinking

The animals were given general anesthesia, and the supertemporal quadrants of the right eyes were exposed. ParaCel (a formula containing 0.25% riboflavin, hydroxy propyl methylcellulose, ethylene diamine tetra acetic acid, trimethyl methylamine, and acetic acid *n*-butyl ester) was applied to completely cover the supertemporal conjunctiva. This process was repeated every 90 s for 4 min. The conjunctival surface was flushed thoroughly with VibeX Xtra (a formula containing 0.25% riboflavin and hypotonic saline). Sufficient VibeX Xtra was applied to completely cover the supertemporal conjunctival surface, and this process was repeated every 90 s for 6 min. The conjunctiva was rinsed completely with a balanced salt solution (BSS). An ultraviolet-irradiation apparatus was used to facilitate a rapid transepithelial CXL treatment. The treatment plan was to use an irradiation intensity of 45 mW/cm^2^, an irradiation spot diameter of 9 mm, a pulse irradiation mode with an interval of 1:1 s, and a total irradiation time of 320 s to obtain a total irradiation energy of 7.2 J. The conjunctiva was rinsed completely with BSS. After the irradiation procedure, levofloxacin eye drops (0.05%) were administered [8,9].

### 2.3 Electron microscopy

The treatment method proposed by Karl et al. [10] was used to observe the collagen after rabbit conjunctiva.

### 2.4 Immunohistochemistry

After removal, the eyes were soaked for 24 h in 4% paraformaldehyde phosphate-buffered saline (PBS). Each eye was dissected along the corneal limbus under a microscope, and the anterior segment was removed after dehydration using the sucrose gradient method. An optical cutting temperature compound (Tissue Tek, Sakura, Japan) was used to freeze the microtome-processed serial section to the treatment conjunctiva (thickness: 10 µm). After drying for 24 h at room temperature, the samples were preserved in a refrigerator at 4°C.

### 2.5 IHC staining procedure

The sample sections were removed from the freezer and placed for 30 min at room temperature. Then, they were immersed in acetone at 4°C for about 10 min and washed with PBS for 5 min three times. The samples were incubated for 10 min in 3% hydrogen peroxide to eliminate enzymatic activity, and they were washed twice with PBS for 5 min each time. Then, a 5% goat serum (PBS dilution) was used to seal the samples, and they were incubated for 10 min at room temperature. The serum was removed without washing, and the samples were dropped into the primary antibody (1:125) dilution, where they remained overnight at 4°C. The next day, the samples were washed in PBS for 5 min three times before being dropped into the secondary antibody, which was marked with biotin (1:125 1% bovine serum albumin in PBS dilution); then, they were incubated for 20 min at 37°C. The samples were washed with PBS for 5 min three times over; then, they were dropped into streptavidin marked with horseradish peroxidase (PBS dilution) and incubated for 20 min at 37°C. They were washed with PBS for 5 min three times and placed into a color-developing agent (3,3 *N*-diaminobenzidine tetrahydrochloride) or 3-amino-9-ethyl carbazole. The samples were fully washed using running water and dyed again. Finally, the film was sealed, and images were taken.

### 2.6 Histology

After the rabbits were euthanized, the treated area was positioned with 10-0 nonabsorbable sutures. Subsequently, enucleation of the eye was performed on each animal, and the eyes were immersed in neutral buffered formalin for 2 days. Subsequently, 2 × 4-mm strips were cut for histological analysis, including from the conjunctiva and sclera. All the sections were subsequently embedded in paraffin. To compare the histological changes, 4-µm-thick paraffin sections of the conjunctiva and sclera were prepared for staining with hematoxylin and eosin (HE). A terminal deoxynucleotidyl transferase-mediated biotin nick-end labeling (TUNEL) assay was performed using a TUNEL assay kit (Promega, USA) according to the manufacturer’s instructions.

The expression of collagen I and collagen III is a reliable marker of fibroblast proliferation. To evaluate this, mouse monoclonal anti-collage antibody (1:100 dilution in PBS) (Merck) was used along with biotinylated goat anti-mouse IgG (code B-6398; Sigma-Aldrich) as the secondary antibody according to the manufacturer’s instructions. All sections were examined using an Axioplan 2 imaging system (Zeiss). Five fields were randomly selected from each section at a magnification of 400× for histological evaluation [11].

### 2.7 Data analysis

As per the previous description by Young [12], longitudinal (filamentous), frontal (circular), and oblique (ellipsoidal) profiles of collagen fibril bundles were found, depending on the orientation within the images.

Because the research objectives are the same, the same analytical method as that of Karl et al. [10] was adopted to measure the diameter of collagen fibers. The method is explained as follows:

To obtain the orientation of the independent diameters of the fibrils, the shortest diameter of each fibril profile was measured. Assuming that the distribution of the collagen fibrils in one bundle was equal in each part of the bundle, fibril distribution patterns were generated from the diameters of 100 neighboring fibrils in one fibril bundle measured using Image-Pro Plus 6.0 analysis software. We used the counting function of the software, which indicates the measured distances automatically with consecutive numbering. Measured distances were automatically transferred into corresponding Excel datasheets and processed as described below. Fibril diameters were plotted against the fibril incidence, and normal distribution fits were made using the IBM SPSS Statistics 19 platform. The data are presented as mean ± standard deviation. For the fibrils’ data, bar charts were generated. Significance was determined with a *T*-test and was accepted at *P* < 0.05. The density and the collagen content of the fibrils were evaluated in images acquired by STEM, and the number of collagen fibrils per μm^2^ was determined using Analysis and Prism software. The collagen fraction (the relative area filled with dark collagen fibrils within the bright interfibrillar matrix) was evaluated with custom-made software that recognized brightness thresholds.

## 3 Results

### 3.1 Ultrastructure of the rabbit conjunctiva

**Figure 1:**
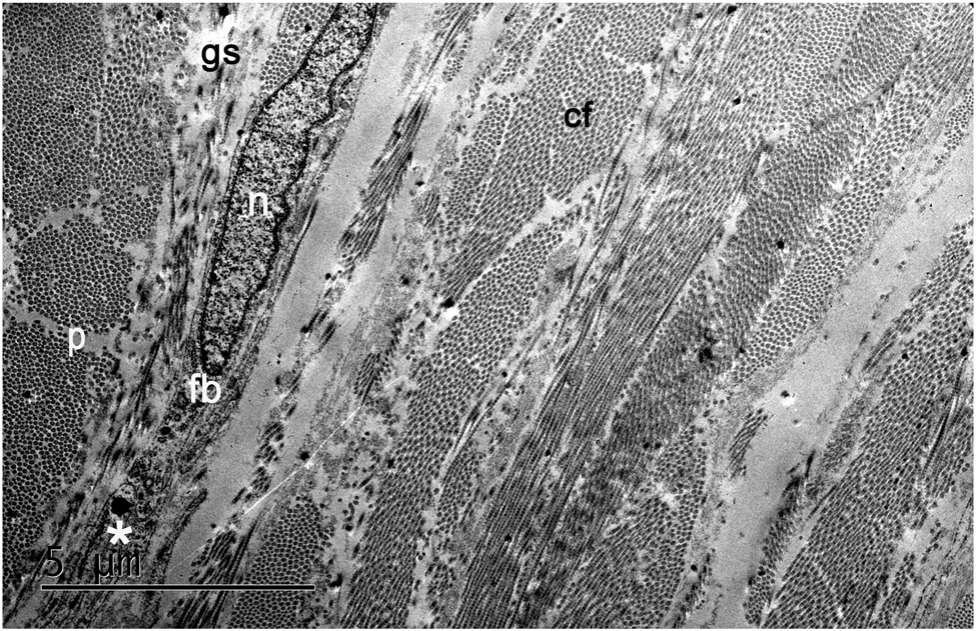
The electron microscopy image of the conjunctiva displays fibroblasts (fb), which are embedded in the interfibrillar matrix (gs: ground substance) and collagen fibril bundles (cf). Fibroblasts display an elliptical nucleus (n) and thin cytoplasmic processes (p), which run parallel to the surface of the eye. Fibroblasts in the conjunctiva contain substantial amounts of rough endoplasmic reticulum (*).

Collagen fibril bundles were present in the conjunctival stroma. The fibril bundles were long and appeared to be interwoven (Figure 1). After conjunctival crosslinking with riboflavin and irradiation with UVA light at 45 mW/cm^2^ for 6 min, the ultrastructure of the conjunctival stroma and the morphology of the cells in these layers showed no difference between the treated and control tissues (not shown). The fibroblasts exhibited distinct signs of cellular activation and degeneration, including cell process thickening, dilation of the endoplasmic reticulum, and cytoplasmic vacuoles (not shown). In addition, erythrocytes in the extracellular matrix indicated the presence of hemorrhage (not shown).

### 3.2 Thickness of collagen fibrils

To determine whether conjunctival crosslinking induces alterations in the thickness of conjunctival collagen fibrils, we measured the diameter of single collagen fibrils in the conjunctival stroma. Note that 100 neighboring fibrils were measured to calculate their diameter, whereas the density of the fibrils described the number of corresponding fibrils per μm^2^ area. The collagen fraction indicated the relative area filled with collagen fibrils within the interfibrillar matrix.

**Figure 2:**
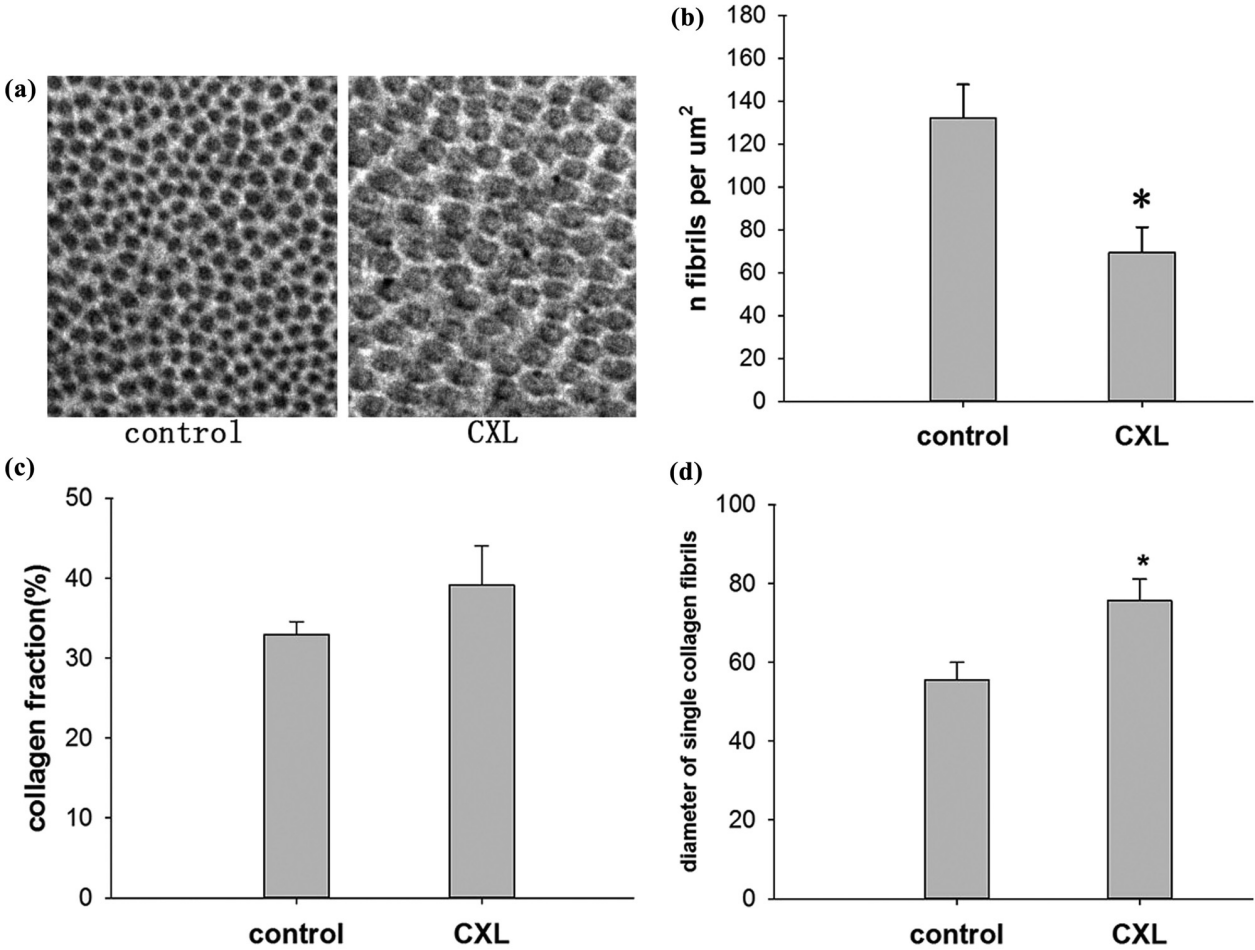
(a) Examples of collagen fibril profiles in electron microscopy images of the conjunctiva of the control eyes and the eyes treated with UV light at an intensity of 45 mW/cm^2^. (b) Number of collagen fibrils per μm^2^ in the fibril bundles of the conjunctiva of the control eyes and the eyes treated with UV light at an intensity of 45 mW/cm^2^. (c) Collagen fraction (the relative area filled with collagen fibrils within the interfibrillar matrix of the conjunctiva) of the control eyes and the eyes treated with UV light at an intensity of 45 mW/cm^2^. (d) The diameter of single collagen fibrils in the control eyes and the eyes treated with UV light at an intensity of 45 mW/cm^2^ (*P < 0.05).

The diameter of the collagen fibrils in the fibril bundles varied slightly and ranged from 30 to 60 nm in the control group of the conjunctival stroma (Figure 2a). In the treatment group, the diameter of the collagen fibrils ranged from 60 to 90 nm (Figure 2a). The thickest collagen fibrils were observed in the treatment group, with fibril diameters measuring up to 90 nm, whereas the thickest collagen fibrils in the control group were considerably smaller (up to 60 nm in diameter). Both collagen fibril groups displayed a unimodal distribution in thicknesses.

The densities of the collagen fibrils were 117.33 ± 5.2 fibrils per μm^2^ and 79.57 ± 5.3 fibrils per μm^2^ in the control and treatment groups, respectively (Figure 2b). The collagen fractions were 0.33 ± 0.01 and 0.39 ± 0.02, respectively (Figure 2c). As shown, treatment with riboflavin and UVA light at 45 mW/cm^2^ significantly increased (*P* < 0.01 and *P* < 0.001, respectively) the diameter of the collagen fibrils in the conjunctival stroma compared with the control group (Figure 2d) and strongly decreased the density of the collagen fibrils in the fibril bundles (Figure 2b). In addition, the collagen fraction (the relative area filled with collagen fibrils within the interfibrillar matrix of the conjunctival stroma) was not significantly increased (*P* > 0.05) after treatment with riboflavin and UVA light at 45 mW/cm^2^.

### 3.3 Changes in collagen fibrils in the rabbit conjunctiva

**Figure 3:**
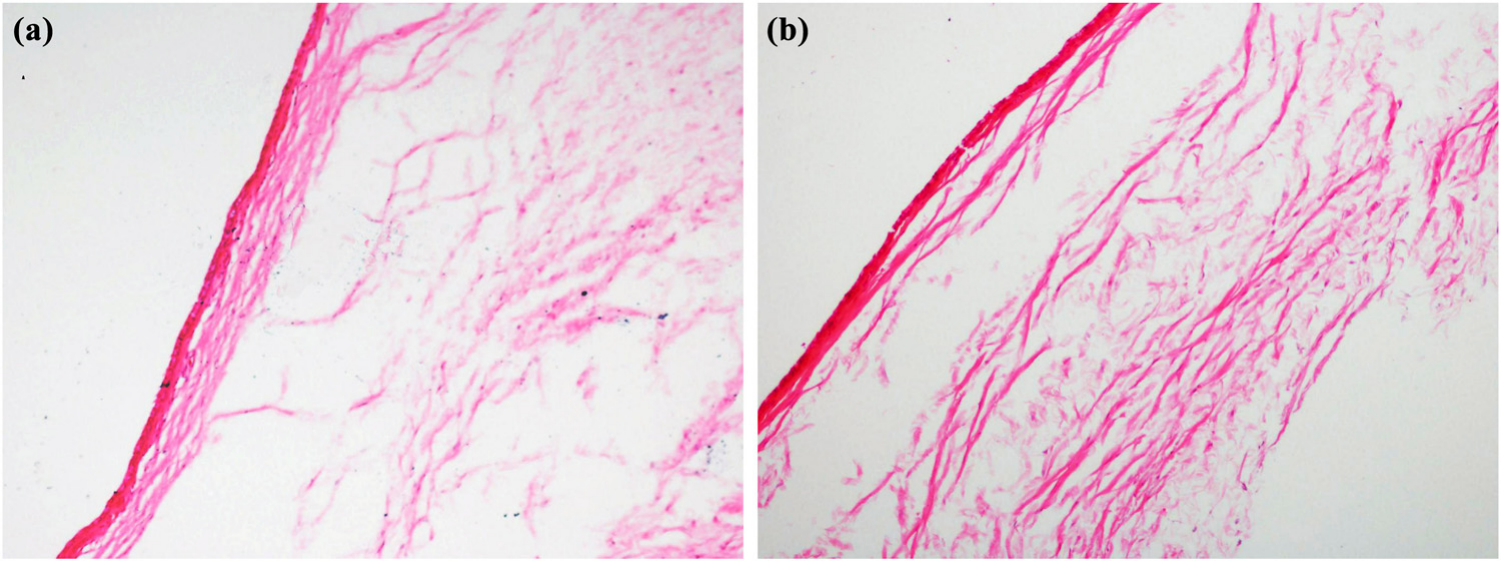
HE staining of conjunctival collagen fibers in control group and treatment group (400×, (a) control group; (b) treatment group).

**Figure 4:**
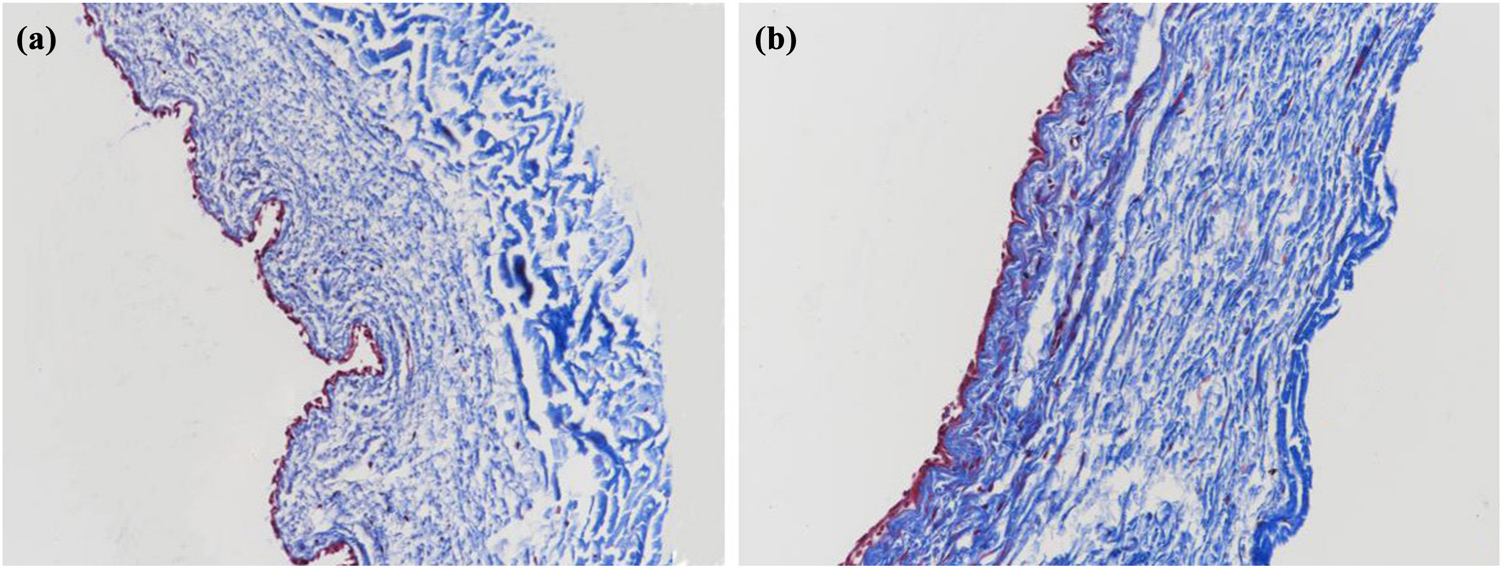
Masson staining of conjunctival collagen fibers in the control group and treatment group (400×, (a) control group; (b) treatment group).

To detect the change in collagen fibrils in the rabbit conjunctiva, HE and Masson tissue staining techniques were used. In Figures 3 and 4, we can see that more collagen fibrils were found in the treatment group compared with those in the control group, and the collagen fibers in the treatment group were arranged more tightly with thicker fibers.

### 3.4 Changes in collagen I and collagen III in the rabbit conjunctiva

In our study, the frozen sections were evaluated using IHC staining. Collagen I and collagen III were observed in both the control and treatment groups. The positive staining of collagen I and collagen III was located outside the cell and presented a brown color. A semi-quantitative analysis of IHC staining results was conducted using Image-Pro Plus 6.0 software.

**Figure 5:**
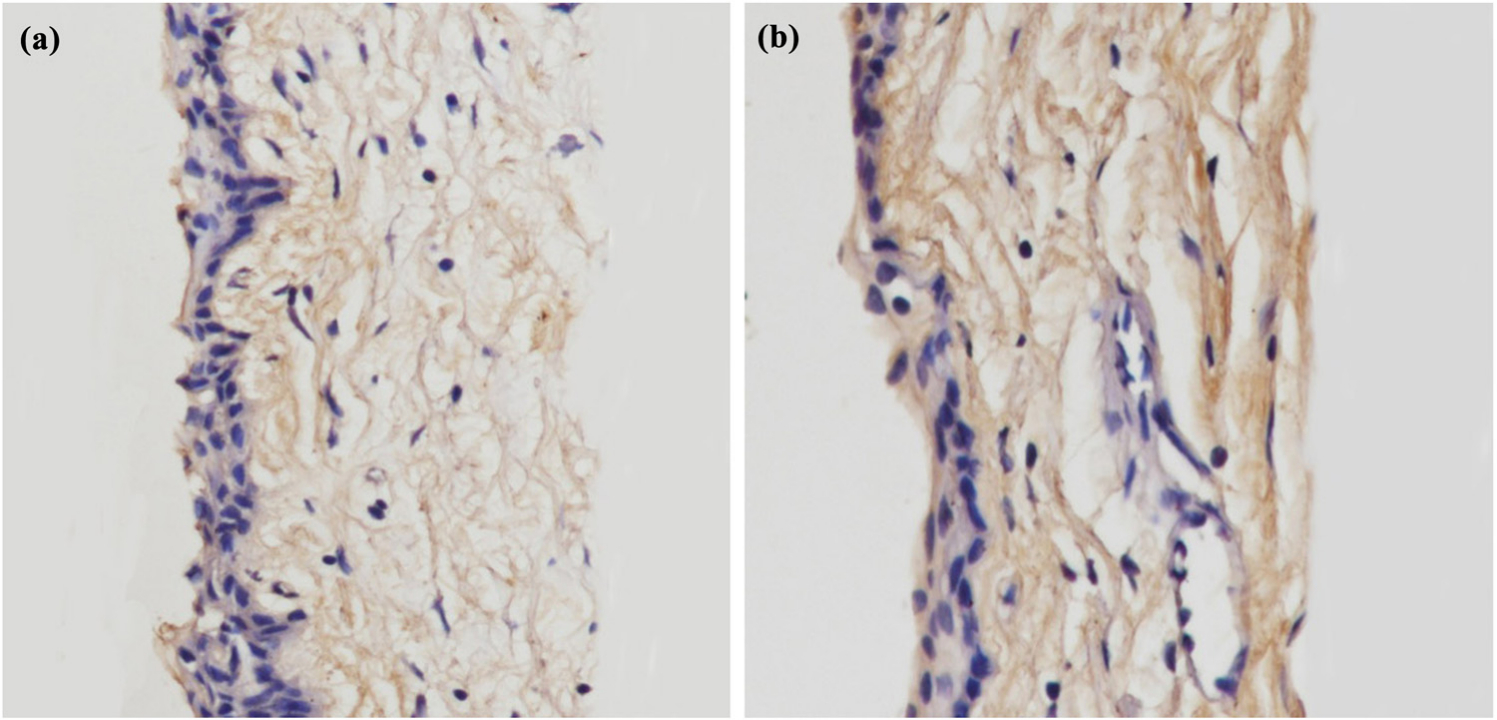
IHC staining of frozen sections. Collagen I was found in the rabbit conjunctiva. When compared with the control group, more collagen fibers were found in the in the treatment group: (a) control group; (b) treatment group.

**Figure 6:**
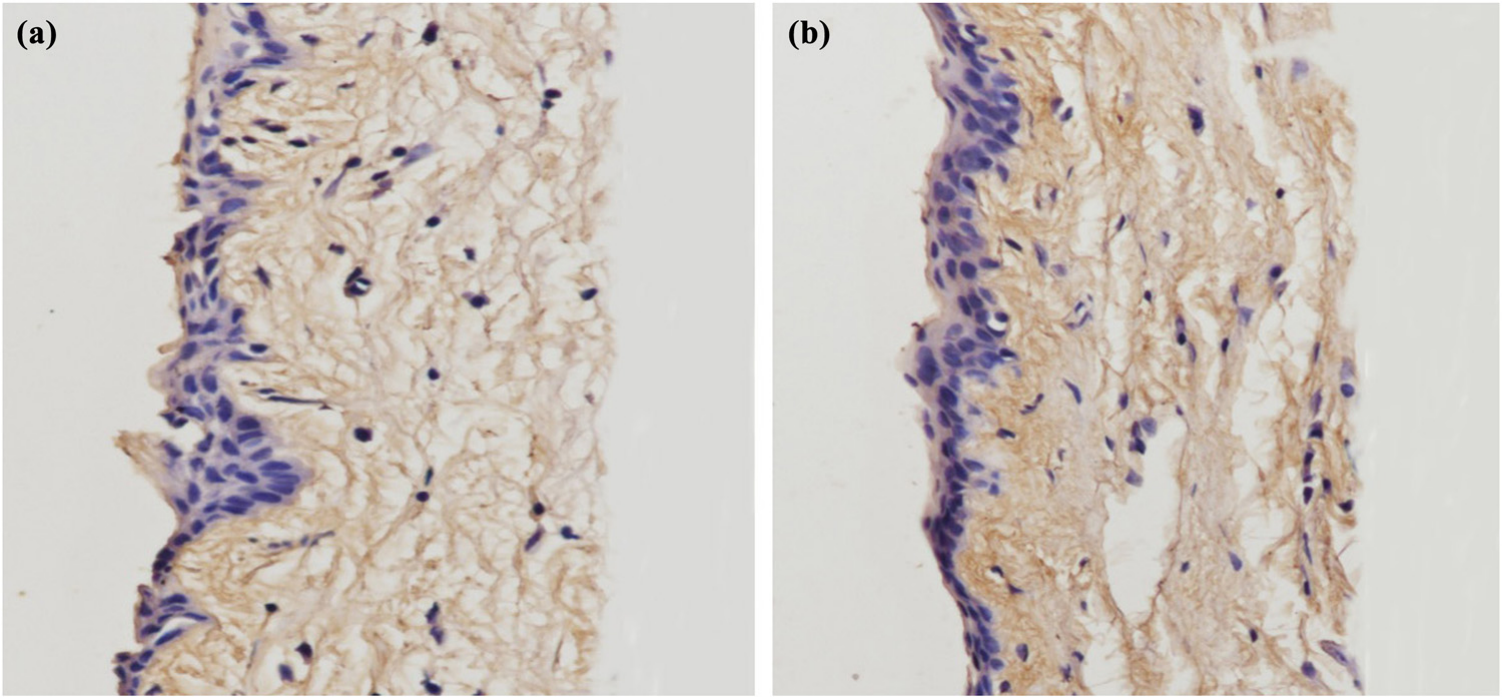
IHC staining of frozen sections. Collagen III was found in the rabbit conjunctiva. When compared with the control group, more collagen fibers were found in the in the treatment group: (a) control group; (b) treatment group.

**Figure 7:**
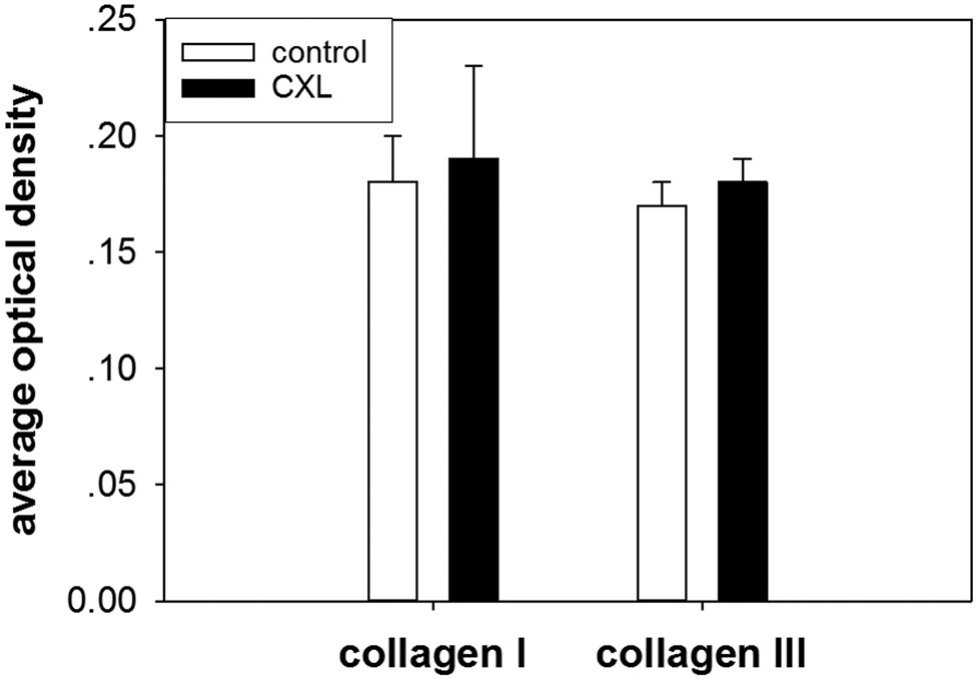
When comparing the control group, the average optical density of collagen I and collagen III were not significantly increased after treatment with riboflavin and UVA light at 45 mW/cm^2^ (*P* > 0.05).

The average optical densities of collagen I were 0.18 ± 0.02 and 0.19 ± 0.04 in the control and treatment groups, respectively (Figure 5). The average densities of collagen III were 0.17 ± 0.01 and 0.18 ± 0.01 in the control and treatment groups, respectively (Figure 6). In our study, average optical densities of both collagen I and collagen III were not significantly increased (*P* > 0.05) after treatment with riboflavin and UVA light at 45 mW/cm^2^ (Figure 7).

## 4 Discussion

CXL was used to increase the tension and stability of the collagen fibers, which causes intra- and inter-molecular covalent bonding. The principle is to use a UVA wavelength of 370 nm to irradiate the tissue infiltrated by the photosensitizer riboflavin. The riboflavin molecule is excited to a triplet state, resulting in an active oxygen species dominated by singlet oxygen [13,14]. Reactive oxygen species can react with various molecules and induce chemical crosslinking reactions between the amino groups of collagen fibers, thereby increasing the mechanical strength of collagen fibers and their ability to resist protease digestion.

This method is currently used to treat keratoconus. The pathogenesis of CCh is similar to that of keratoconus [15]. The type, number, and spatial structure of collagen are changed, and its mechanical tension is reduced. In this study, we determined the ultrastructural changes in cells and collagen fibrils in the rabbit conjunctiva after conjunctival treatment with riboflavin and irradiation with UV light.

We found that conjunctival crosslinking with riboflavin and irradiation with a UV light intensity of 45 mW/cm^2^ for 6 min influenced the amount and diameter of the collagen fibrils in the bundles of the conjunctival stroma. The fibroblasts showed distinct signs of cellular activation, and the presence of erythrocytes in the conjunctival extracellular matrix indicated the existence of hemorrhage (not shown). It cannot be excluded that the changes observed under the conditions used in this study were not caused by light irradiation but by the surgical procedure itself. The results suggest that conjunctival crosslinking with riboflavin and UV light at 45 mW/cm^2^ for 6 min is safe and does not induce various inflammatory or degenerative processes in the conjunctiva and adjacent tissues.

It has been suggested that the biomechanical properties of the conjunctiva result from the diameter, distribution, and orientation of collagen fibrils [16,17]. We found that the diameter of collagen fibrils in the conjunctival stroma varied slightly from 30 to 60 nm in the control group and from 60 to 90 nm in the treatment group. This range is similar to the data described in a previous study [12]. However, we found that the diameter of the collagen fibrils exhibited a unimodal distribution.

In this study, we detected the changes in collagen fibrils in rabbit conjunctiva by HE and Masson tissue staining. In Figures 3 and 4, we can see that more collagen fibrils were found in the treatment group compared with those in the control group. The collagen fibers in the treatment group were arranged more tightly, and the fibers were thicker.

In our experiment, we also applied frozen-section IHC staining to detect collagen I and III expressions in the rabbit conjunctiva by changing the conjunctival morphology. We observed that the positive staining of collagen I and collagen III was located outside the cell and presented a brown color. Average densities of both collagen I and collagen III were not increased after treatment with riboflavin and UVA light at 45 mW/cm^2^. CCh is associated with a thinning of the conjunctival collagen fibrils, which may be a reason for the decrease in conjunctival stiffness and thinning [18,19,20]. We found that treatment with riboflavin and UVA light at an intensity of 45 mW/cm^2^ caused a significant increase in the diameter of the collagen fibrils in the fibril bundles. In the conjunctiva, activated fibroblasts and macrophages may produce collagen-degrading exoenzymes.

The results of this study may indicate that crosslinking with riboflavin and UV light results in a remodeling of the conjunctival extracellular matrix, which includes the degradation of thick collagen fibers and/or the *de novo* synthesis of collagen fibers [21,22]. However, there was temporal damage to the conjunctival cells after conjunctival crosslinking using riboflavin and UVA light. Delayed resettlement of conjunctival cells in the treated tissue leads to prolonged regeneration processes and long-lasting biomechanical stiffening effects [12]. Conjunctival remodeling may also contribute to scarring and stiffening. Extensive conjunctival light irradiation should be avoided due to the risk of tissue damage in the choroid and retina. Advanced technologies using high light intensities and minimized irradiation spots (similar to spot welding) can use the benefits of such remodeling side effects to stiffen the conjunctiva and prevent tissue damage to the retina.

## 5 Conclusion

To increase the safety of the conjunctival crosslinking method, developing different therapeutic approaches to prevent the harmful effects of high-intensity light treatment without inhibiting crosslinking efficiency may be helpful. In addition, the topical administration of anti-inflammatory agents may help prevent chronic inflammation.
